# The contribution of breastfeeding to a healthy, secure and sustainable food system for infants and young children: monitoring mothers’ milk production in the food surveillance system of Norway

**DOI:** 10.1017/S1368980022001495

**Published:** 2022-10

**Authors:** Julie P Smith, Britt Lande, Lars Johansson, Phillip Baker, Anne Bærug

**Affiliations:** 1 College of Health and Medicine, Australian National University, Canberra 2601, Australia; 2 Division for Prevention and Public Health, Norwegian Directorate of Health, Oslo, Norway; 3 Institute for Physical Activity and Nutrition, Deakin University, Geelong, Australia; 4 Norwegian Institute of Public Health, Oslo, Norway

**Keywords:** Food systems, Food surveillance, Infant and young child nutrition, Breastfeeding, Infant formula, Human milk

## Abstract

**Objective:**

The mother–child breastfeeding dyad is a powerful force for achieving healthy, secure and sustainable food systems. However, food system reports exclude breastfeeding and mother’s milk. To help correct this omission and give breastfeeding women greater visibility in food systems dialogue and action, we illustrate how to estimate mother’s milk production and incorporate this into food surveillance systems, drawing on the pioneering experience of Norway to show the potential value of such analysis.

**Design:**

The estimates use data on the proportion of children who are breastfed at each month of age (0–24 months), annual number of live births and assumptions on daily human milk intake at each month. New indicators for temporal and cross-country comparisons are considered.

**Setting:**

It is assumed that a breastfeeding mother on average produces 306 l of milk during 24 months of lactation.

**Participants:**

The annual number of live births is from Statistics Norway. Data for any breastfeeding at each month of age, between 0 and 24 months, are from official surveys in 1993, 1998–1999, 2006–2007, 2013 and 2018–2019.

**Results:**

Estimated total milk production by Norwegian mothers increased from 8·2 to 10·1 million l per year between 1993 and 2018–2019. Annual per capita production increased from 69 to 91 l per child aged 0–24 months.

**Conclusions:**

This study shows it is feasible and useful to include human milk production in food surveillance systems as an indicator of infant and young child food security and dietary quality. It also demonstrates significant potential for greater milk production.

The mother–child breastfeeding dyad is a powerful force for achieving healthy, secure and sustainable food systems. It represents a ‘first-food system’ in and of itself being paradoxically both a globally distributed form of food production^(1,2)^, and yet the world’s shortest, most localised food supply chain. When supported, breastfeeding women deliver ‘round the clock’ food on an equitable basis^(3)^, as well as providing nurturing care to infants and young children. Norway is unusual among high-income countries as virtually all Norwegian mothers initiate breastfeeding, and the country counts their milk production as part of the country’s food supply statistics. Our purpose is to show the potential value of collecting and analysing this data, using Norway as an example.

Breastfeeding provides a healthy start to life. As the biological ‘first-food’ for humans, the milk provided by the world’s breastfeeding mothers is a safe and nutritionally optimised food that is uniquely responsive to children’s evolving nutritional needs. Breastfeeding provides immunological and other factors which support healthy growth and development throughout the life course of the child as well as the health of the mother. Good evidence from a variety of country settings shows that not breastfeeding increases children’s risk of all-cause mortality, diarrhoea, respiratory infection and malocclusion, and likely obesity and type 2 diabetes in later life^(4)^. The child who breastfeeds in early life is more likely to achieve their full cognitive potential, and hence perform better at school and work in later life, which translates into higher productivity for wider society^(5)^. For mothers, insufficient breastfeeding heightens the risk of breast cancer, and likely ovarian cancer and type 2 diabetes^(4)^. Breastfeeding also helps with birth spacing and benefits women’s reproductive health. Every year, over 820 000 child deaths from pneumonia and diarrhoea, nearly 1 million cases of child obesity, and 100 000 maternal deaths from cancers and type 2 diabetes can be attributed to not breastfeeding^(4)^. Society also benefits economically from high breastfeeding prevalence, as it helps reduce health treatment costs and avoid excess morbidity and mortality^(6)^.

Despite the importance for early nutrition and public health, major food systems reports do not include breastfeeding women and their production of human milk. This is a remarkable omission, given that infants and young children (0–36 months) comprise 6% of the world’s population and consume significant amounts of breastmilk as a staple food source in all country contexts. Multi-country estimates show that breastfeeding contributes all the breastfed child’s energy needs during the period of recommended exclusive breastfeeding and about 35% in the second year of life when breastfeeding is continued^(7)^. Arguably, food systems and monitoring frameworks that ignore breastfeeding will fail to identify important aspects of children’s interaction with the food system or key gaps and opportunities to address children’s dietary needs.

The WHO recommends initiating breastfeeding within the first hour after birth, thereafter exclusive breastfeeding for the first 6 months of life and introduction of nutritionally adequate and safe complementary (solid) foods at 6 months together with continued breastfeeding up to 2 years of age or beyond^(8)^. Yet fewer than half of infants and young children worldwide meet these WHO recommendations for optimal nutrition. Whereas exclusive breastfeeding at the age of 6 months has increased from 35 % in 2005 to 42 % by 2019, and any breastfeeding at the age of 1 year has increased somewhat in high-income countries, continued breastfeeding has decreased in low-middle income countries and around 45 % of children are breastfeeding at the age of 2^(9,10)^.

In contrast, recent studies show sales of commercial milk formula products, including infant formulas and toddler milks, are growing markedly^(1)^. This trend is expected to continue, raising serious concern for global child and maternal health and further compounded by the harmful impacts of the COVID-19 pandemic on breastfeeding and early-life nutrition^(11)^. This global infant and young child transition towards a diet dominated by commercial ultra-processed foods^(12)^ highlights that breastfeeding is a vulnerable human food system. Existing policy and regulatory frameworks designed to protect, promote and support breastfeeding are currently failing to adequately protect child nutrition against continued global industry expansion^(1)^.

Exclusive breastfeeding is one of WHO’s ‘Double Duty Actions for Nutrition’ to tackle the double burden of undernutrition and overweight, obesity and diet-related non-communicable diseases. Improving breastfeeding can also directly address food system sustainability and the relevant Sustainable Development Goals^(13)^. It is now well recognised that structural and cultural contexts, health services, commercial influences, family and workplace settings, as well as individual factors shape breastfeeding practices^(5)^, emphasising the important role of policies to protect, promote and support breastfeeding^(8)^. Breastfeeding policies, programmes and investments framed by the 2003 WHO/UNICEF Global Strategy on Infant and Young Child Feeding is an important but neglected element of strategies for sustainable food systems^(13)^. Well established, effective interventions on breastfeeding are available for integrated policy actions in all country settings^(14)^. Strengthening monitoring systems that track the progress of policies, programmes and funding towards achieving both national and global breastfeeding targets is one of the seven policy actions in the 2015 Call for Action by the Global Breastfeeding Collective, led by UNICEF and WHO.

To help correct the omission of human milk production in food systems monitoring frameworks and to give the food productivity of breastfeeding mothers greater visibility within food systems thinking, dialogue and action, we illustrate how this milk production can be estimated and incorporated in national and international food surveillance systems, using the example of Norway.

Norway has pioneered the measurement of human milk production and its inclusion into national food statistics and has been influential internationally in this regard. Estimates of milk production by Norwegian women (‘produksjon av morsmelk’) have now been present in Norway’s food statistics for over two and a half decades^(15,16)^. The terminology ‘mothers’ milk highlights and acknowledges women’s productivity and contributions to food supply for infants and young children and reflects its translation from the Norwegian word *morsmelk* in Norway’s official documents reporting its production. In this article, we use the term ‘human milk’ consistent with terminology in economic statistics and food surveillance systems that report on commercial milk products from other species, and ‘milk’ refers to ‘mothers’ milk’, ‘breastmilk’ or ‘human milk’.

A study in 1973 by a nutrition economist from the World Bank was the first to draw attention to the adverse economic and food supply implications of global declines in breastfeeding^(17)^. Around this time, Norwegian delegates to the FAO of the UN sought to include breastfeeding in indicators of countries’ national food supply, and Norway was the first high-income country for which such estimates were made^(18)^. Australia was the second^(19)^.

Norway also has comprehensive and long-established policies in place to enable breastfeeding^(20)^. The Norwegian National Nutrition Council anticipated as early as the 1970’s that including human milk production in food statistics could serve to recognise its significance and motivate policies to protect breastfeeding^(18)^. Studies of human milk production since that time have similarly been motivated variously by the desire to provide better scientific information for public policy and budgeting decisions, reduce the invisibility of women’s productivity, including breastfeeding and draw attention to the need for measures to prevent or address declines in breastfeeding. Breastfeeding rates are high in Norway compared with other high-income countries^(21)^. Nearly all mothers initiate breastfeeding and around half continue to do so at 12 months. Yet few children breastfeed exclusively for 6 months or breastfeed to 2 years or longer.

The aim of this study is to review trends in estimates of human milk production for infants and young children (0–24 months) in Norway between 1993 and 2018–2019 and to discuss and present this method for monitoring human milk production in national food statistics. We extensively discuss and assess key issues in such methodologies and elaborate further on the Norwegian experience of monitoring human milk production.

## Methods

To address the study objective, we adopted the approach of reviewing all estimates of Norwegian human milk production since 1993, involving the following steps:

- Firstly we describe the key data inputs and illustrate how these are used to make the estimates;
- Secondly, we present summary data for annual estimates of human milk production drawing from successive reports for the period 1994–2020 and report calculations of the average per capita amounts of milk that mothers supplied each year for children aged 0–24 months; and
- Finally, we assess the methods and assumptions used to estimate the production figures.

A table on human milk production is published in annual reports on trends in the Norwegian diet from the Norwegian Directorate of Health. The calculations have been made in a collaboration between the Norwegian Directorate of Health and the Norwegian National Advisory Unit on Breastfeeding. These official estimates of human milk production in Norway use the method developed and published by Oshaug and Botten in 1994^(18)^. This is summarised below.

The estimation is based on data for annual number of live births, proportion of any breastfeeding at each month of age and daily human milk intake per breastfeeding child at each month of age.

### Annual number of live births

The annual number of live births is provided by Statistics Norway from its basic collections^(22)^. As the number of deaths of children aged 0–24 months is very low in Norway, the number of live births is taken to be the number of infants (0–12 months).

For simplicity, the same number of live births is also used as the number of toddlers (13–24 months), that is, children born in the previous year.

### Proportion of breastfeeding

Calculations on human milk production use data on any breastfeeding at each month of age, for children 0–24 months. This was provided from the following surveys covering breastfeeding practices in Norway: data for 1993 from records in community health services in five counties^(15)^, three national dietary surveys among infants and young children (in 1998–1999, 2006–2007, 2018–2019)^(23–31)^ and one national survey on breastfeeding in 2013^(32)^.

Further information on Norway’s data sources on births and dietary surveys for the 0–24 months age category is provided in Supplementary Table 1.

### Estimates on daily human milk intake per breastfeeding child

The daily intake used by the Norwegian Directorate of Health to calculate national human milk production for the 0–24 months age group is based on data from the study by Oshaug and Botten (1994) with some minor adjustments made on the basis of a 2002 WHO report on human milk intake^(33)^. After such adjustments, the calculations assume that Norwegian mothers who breastfeed the baby throughout the first year of life will on average produce 225 l of milk, and breastfeeding in the second year will produce a further 81 l, for a total of 306 l of human milk during a lactation period of 0–24 months^(16)^. Comparative human milk intakes from other studies are summarised in Supplementary Table 2.

Table 1 illustrates these calculations of human milk production in Norway, using the most recent data^(16)^.

**Table 1:** Human milk production in Norway 2018–2019

Child age (months)	Proportion of children breastfeeding (%)*	Number of children breastfeeding per month†	Volume of human milk intake per child per day (l)‡	Volume of human milk intake per child per month (l)§	Production of human milk (ml)||
1	93	51 262	0·7	21	1·077
2	89	49 057	0·7	21	1·030
3	85	46 852	0·8	24	1·124
4	82	45 198	0·7	21	0·949
5	79	43 545	0·7	21	0·914
6	78	42 994	0·7	21	0·903
7	72	39 686	0·6	18	0·714
8	68	37 482	0·6	18	0·675
9	63	34 726	0·6	18	0·625
10	58	31 970	0·5	15	0·480
11	51	28 111	0·5	15	0·422
12	48	26 458	0·4	12	0·317
First year of life (0–12 month)					9·230
13	34	18 741	0·3	9	0·169
14	29	15 985	0·3	9	0·144
15	24	13 229	0·3	9	0·119
16	21	11 575	0·2	6	0·069
17	20	11 024	0·2	6	0·066
18	15	8268	0·2	6	0·050
19	14	7717	0·2	6	0·046
20	12	6614	0·2	6	0·040
21	11	6063	0·2	6	0·036
22	10	5512	0·2	6	0·033
23	9	4961	0·2	6	0·030
24	8	4410	0·2	6	0·026
Second year of life (13–24 month)					0·828
Total per year					10·058

Source^(16)^: with minor revision.

* Any breastfeeding, data from national dietary surveys among infants and young children (Spedkost and Småbarnskost (2018–2019). See references in text.

† Proportion of infants or young children breastfeeding each month multiplied by number of live births in 2018: 55 120.

‡ Estimates of daily human milk intake, based on Oshaug and Botten (1994) and minor adjustments according to the WHO study (Butte 2002).

§ Volume of human milk intake per child per month: volume of human milk per day multiplied by 30 d.

|| Production of human milk per month: number of children breastfeeding at each month of age multiplied by volume per child per month.

From the outset, it was recognised that the calculations rely on several assumptions and simplifications. Notably, as shown in Table 1, the estimates do not differentiate for whether the child is exclusively breastfeeding or not; hence the milk intake per day of 700–800 ml for a breastfeeding child is an approximation for the first 6 months. From 6 months, the amount of complementary food can replace breastmilk to a varying extent. Therefore, the calculated production can only indicate the size range. There have been only minor changes in these assumptions since the beginning of these estimations.

## Results

The estimated total production of human milk for 0–24-month-old children in Norway increased from 8·2 to 10·1 million litres per year between 1993 and 2018–2019 (Table 2)^(15,34,35)^. The largest increase was seen during the 1990’s. After 2006–2007, the estimated milk production has fluctuated, with a decrease in 2013^(36)^ and a small increase in 2018–2019^(16)^.

**Table 2:** Human milk production in Norway 1993–2019*

	1993^(15)^	1998–1999^(34)^	2006–2007^(35)^	2013^(36)^	2018–2019^(16)^
Total volume per year (millions of litres)	8·2	10·3	10·5	10·0	10·1
Volume per capita per year (l)†	69	88	90	83	91

* The calculations are based on average amounts of human milk production for children aged 0–24 months.

† This calculated as annual milk production divided by the number of children aged 0–24 months in that year.

When considering the variable number of births in this period, the average per capita volume of human milk per child (all children aged 0–24 months) per year increased from 69 l in 1993 to 91 l in 2018–2019, with a transient decline in 2013 (Table 2). This represents an overall increase in production per capita of around 30%.

**Fig. 1:**
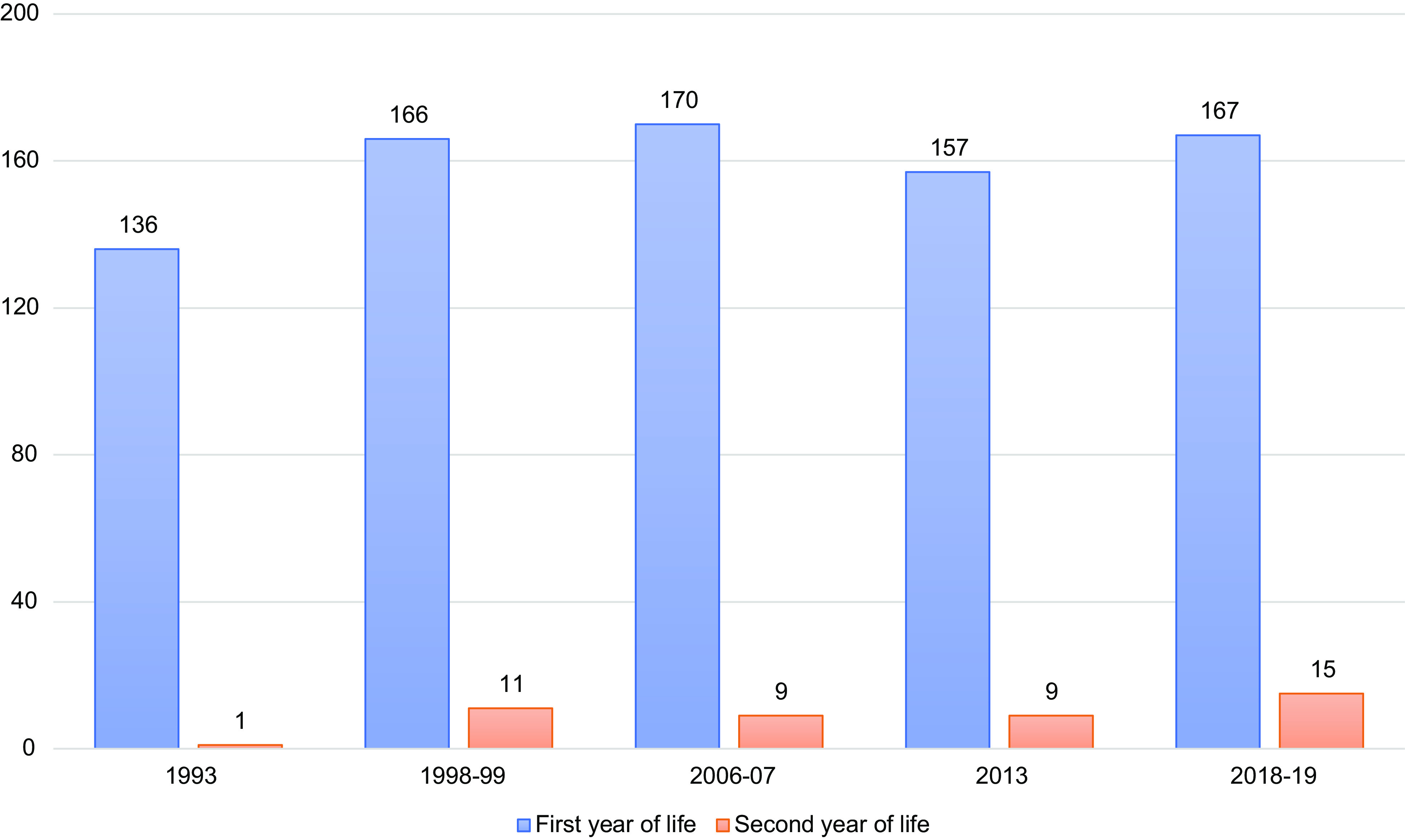
Human milk production in Norway 1993–2019, litres per child 0–24 months *Note*: This differs from the annual per capita figures in Table 2 because this is the amount a Norwegian child receives during the lactation 0–24 months and is separated into the first and second year of life.

Figure 1 shows the average human milk production per child during the lactation period of 0–24 months, separated into the first and second year of life. These figures differ from the annual per capita figures in Table 2. Most of the milk produced (92 % in 2018–2019) was by mothers of infants in the first 12 months of life, from 1993 to 2018–2019. Human milk production has been growing in the second year of life, although it remains at low levels for this age group.

## Discussion

We have shown how estimates of human milk production can be incorporated into national food surveillance systems, using the example of Norway. The estimated total milk production by Norwegian mothers rose from 1993 to 2018–2019 to around 10 million litres a year, showing the large scale of the contribution of breastfeeding for national food supply for infants and young children. A key innovation in this paper is the analysis estimating *per capita* human milk production, enabling comparison of trends and patterns of production within and across countries^(37)^. The rise since 1993 in Norway shows the potential for substantially increased human milk production, particularly if enabling policies and programmes are in place. Analysis of Norwegian data illustrates the potential utility of additional metrics to assist cross-country and intertemporal comparisons and to help relate breastfeeding data to the food system and measures of economic output.

There is already a recognised need for better metrics of how food systems affect nutrition and health^(38)^. Nevertheless, the continued invisibility of breastfeeding as a form of food production and human milk as a food for infants and young children is starkly illustrated in a proposed framework (the ‘Innocenti Framework’) for conceptualising how the food system shapes the diets of children and adolescents^(39)^ and by a recently published framework for comprehensively monitoring transformative food system change^(40)^. The latter calls attention to the need to monitor food systems globally to inform decisions and support accountability for better governance of food systems. Concerningly, both of these important frameworks failed to mention breastfeeding, despite its importance as the ‘first-food system’, or the importance of its adequate monitoring for policy decision-making and accountability for progress.

Structural determinants of breastfeeding need to be addressed by policy to make progress towards global and national nutrition targets^(5)^. A pioneering 1994 Norwegian study of human milk in food supply statistics^(18)^ emphasised that monitoring trends in human milk production helped make visible the importance of policy or programme measures to protect, promote and support breastfeeding. In Norway, monitoring the contribution of mothers’ milk to healthy, secure and sustainable food production is a continuing recognition of the importance of breastfeeding in public policy. Exclusion from economic statistics such as Gross Domestic Product^(41)^ has also been argued to result in incomplete and biased estimates of national food production and overall economic output, and slants policy priorities, to the disadvantage of women and children. The French Presidential Commission on the Measurement of Economic Performance and Social Progress (S-S-F Commission), led by Joseph Stiglitz and Amartya Sen, warned of serious bias and distortion of the economy away from what is most valuable if economic statistics failed to note shifts from home production to market production^(42)^. They cited human milk as an example of the problem:

‘There is a serious omission in the valuation of home-produced goods—the value of breast milk. This is clearly within the System of National Accounts production boundary, is quantitatively non-trivial and also has important implications for public policy and child and maternal health.’

In contemporary perspective, including estimates of human milk in food production statistics not only highlights its value to countries as an economic resource, but also facilitates integrating global and national breastfeeding strategies and policies with current food systems thinking, dialogue and action, and adapting or strengthening such measures when the social and market environment changes. Evidence of a rapid transition towards milk formula consumption in middle-income countries in recent years^(12)^ along with indications of declining breastfeeding indicators in low-income countries and some regions emphasises there is an urgent need for better monitoring of trends as countries develop their market economy^(9)^. Rising incomes, urbanisation, the changing nature of woman’s work, social norms, media influences, medicalisation and globalisation of the baby food industry have been identified as key determinants of this global transition in diets of infants and young children towards commercial milk formula^(1)^.

While the Norwegian estimates analysed in this study have official status, they sit alongside several academic studies since the early 1970’s which have estimated (national) volumes of milk produced by breastfeeding women in various countries. Estimates currently exist for twenty three different countries, dating back to 1908 for the Philippines^(17)^. Studies conducted during the 1980’s and 1990’s in Indonesia and India demonstrated that this was a substantial national food resource^(43,44)^. A study calculated human milk production in sub-Saharan African countries in 1997^(45)^, and further studies of human milk supply at the national level have since been published for India^(46)^ as well as for Bolivia^(47)^, Francophone west Africa^(48)^ and China^(49)^, and four high-income countries (USA, UK, and Australia, and Norway)^(37)^.

However, Norway’s estimates remain the only ones that provide a time series showing trends over time that are calculated on a consistent basis for the 0–24 months age group over more than two and a half decades. The annual per capita human milk production for infants and young children in Norway from 1993 to 2018–2019 was shown in this study to be in the range 69–91 l per child aged 0–24 months. This was higher than in other high-income countries for which estimates are available. Using the estimates from those studies, it can be calculated that annual per capita production was around 64 l per child in Australia in 1992^(19)^, rising to 69 l by 2010 and comparing with 61 l in the USA in 2009 and 90 l in Norway in 2006–2007^(37,50)^. Per capita annual production of around 64–67 l in China^(49,50)^ during the period 2001 and 2008 is similar to the levels in the above high-income countries. In India, it rose from around 72 in 1992–93 to 84 l in 1998^(43)^: more recent estimates are for production of around 137 l per child per year^(46)^. Estimates for Bolivia in 2001 suggest around 110 l per child per year^(47)^ and comparably 100 l for The Philippines in 2008. Production has been much higher in many African countries, up to 150–170 l per child annually for breastfeeding of the 0–36 months age group^(45,48)^. The above illustrates that the per capita measure of human milk production can provide insights into trends over time and differences across countries in food security for infants and young children.

However, there is a need for a common methodology. In these studies, the estimated quantities of milk supplied by mothers varied across countries and within the same countries over time due in part to differences in study scope or method. This can make temporal or cross-country comparisons complex. For example, firstly, the academic studies of human milk production differed in the child age range included in the estimates, which for Norway was 0–24 months. Most studies covered this age range, but a small number covered early infancy only. By contrast, the scope of some estimates was birth to 36 months of age^(44,45,47–49)^. Relatedly, studies also differ in how the number of infants and young children was measured. Most took the number of live births as a reasonable proxy to measure the number of infants and young children in the study and used births for the current year as indicating the number of children living in the second (or in some cases the third) year. Few adjusted for mortality. In Norway, the effect is not large because infant mortality is very low. However, if infant mortality is high, a possible difference between the number of live births and the population of infants and young children may need to be considered in any cross-country or intertemporal comparisons.

Secondly, survey methods on breastfeeding prevalence may also differ, including over time. Survey methods and sampling of data on any breastfeeding in Norway are similar in the national surveys from 1998–1999, 2006–2007, 2013 and 2018–2019, whereas, data for 1993 is from community health services in five of twenty counties^(15,23–31)^. These surveys are broadly representative, but there is some under-representation of immigrants and mothers with lower education in the national surveys. The estimates for Norway use data on any breastfeeding at each month of age and do not distinguish exclusive breastfeeding from partial breastfeeding in the 0–6 months aged group. In 2018–2019, the proportion of exclusively breastfeeding infants in Norway was lower from 4 to 6 months of age than it was in 2006–2007, but the proportion of breastfeeding in the 13–24 month age group was higher than in 2006–2007. Estimates for most other countries likewise used data on any breastfeeding rates by month of age. However, calculations in some studies^(47,48)^ accounted specifically for the prevalence of exclusive breastfeeding.

Thirdly, studies used various assumptions about daily human milk intake per child, depending in part on the extent of exclusive breastfeeding for the 0–6 months population of infants. This highlights the need for common assumptions across estimates for countries and over time. In some studies, the assumed daily intake implied during a 0–24 month lactation period ranged from around 290^(44)^ to around 310^(37,46,50)^ l. This aligns with the 306 l used for official estimates in Norway^(16)^. However, many other studies assumed much higher daily breast milk intake, ranging from an overall intake of 331 l^(18)^ to as high as 443 l^(47)^ for the 0–24 month age range^(17,41,45,51)^. The Norwegian assumption that the first 6 months of breastfeeding provides around 130 l of milk is consistent with other studies (range is108 l^(44)^ to 155 l^(17)^). Also, the Norwegian assumption of 225 l for the 0–12-month period is comparable with the range cited in other studies of around 180–290 l^(33,44)^.

On the other hand, its assumption of milk intake of around 80 l for young children 13–24 months is conservative compared with other estimates (range from 106^(18)^ to 201 l)^(7,47)^. This is considerably lower than the lowest levels in other studies. Thus, Norway’s present estimates may understate production in the 13–24 month age group. There is a paucity of evidence on the intake for children beyond the first year. Findings for Indonesia^(44)^ and studies which calculated milk production for children up to 24–36 months^(45,47–49)^ emphasise that declines in breastfeeding prevalence for children in the second year of life and beyond translate into a substantial loss at country level. Human milk production estimates for Norway in 2018–2019 could be 10–50 % higher (∼11–15 million litres a year) if the higher range of assumed milk intakes (331–443 l) for the 0–24 months age group in other studies of milk production was used. This shows the need for better evidence of milk intake including in traditionally breastfeeding populations.

Norway estimates the amount of actual human milk production in a given year, but several studies have also estimated countries’ quantity or monetary value of ‘lost milk’ compared with the potential from more optimal levels of breastfeeding^(17,19,47,48,50)^. Tracking the extent of ‘lost milk’ using a consistent methodology and assumptions about yields and potentially feasible breastfeeding levels may be a second useful system level approach to measuring a deterioration in food security and nutrition for infants and young children. Lost milk was calculated in most such studies as the difference between the measured level of actual human milk production, compared with a measure of the biological production potential of the mothers. Using a 95% benchmark for breastfeeding rates to define the biologically feasible production level^(41)^, around 18 million litres of human milk would have been available for Norwegian children in 2006–2007, meaning that 40% of potential milk supply in Norway was ‘lost’^(37)^. Recent rapid declines in breastfeeding during the COVID 19 pandemic^(52)^ reinforce that current levels of production of human milk cannot be taken for granted. Continued provisioning through this first food system needs to be supported so that it is not ‘lost’.

Our findings have implications for public policy, including monitoring and surveillance. The method used to estimate human milk production in Norway is applicable to other countries. It requires national data on breastfeeding and number of births. Regular and accurate data on breastfeeding practices for 2 years and beyond, with regular data collections and continuity of survey design and methodologies over time, is crucial^(4)^. Data deficiencies are particularly apparent in high-income countries^(5)^. A recent review called for stronger investments in data for monitoring breastfeeding trends^(21)^.

This research highlights that there are now established concepts and evidence on the value of human milk that could be used more systematically and on a wider scale, such as by developing a tool for use in food surveillance, policy analysis and advocacy. While existing commercial data such as on *per capita* milk formula sales are used by industry to evaluate the size and potential market for these products, data on human milk production are more useful for policymakers evaluating food security and dietary quality of infants and young children.

The omission of human milk from relevant food statistics has an impact on policy decisions of relevance to the health of children and on the status of women, breastfeeding practices and nutrition and health policies.

Demonstrating the amount and significance of human milk production provides evidence supporting its inclusion in food statistics. As stated by Hatløy and Oshaug 25 years ago, central actors in the decision-making process, such as economists, statisticians and others, should be made aware of such facts on human milk production because of the important implications of the quantities produced. This alone justified the inclusion of human milk in food statistics. By not including the production of this nutritionally adequate and health-promoting food, attention to the economic asset would continue to be low. Furthermore, the omission might directly contribute to the decline of breastfeeding and continued discrimination against lactating women and their young children, with serious health consequences. Political support and financial investment are also needed to protect, promote and support breastfeeding^(11)^.

## Conclusions

The milk produced by breastfeeding mothers is a recognised component of the food supply statistics in Norway. The experience of Norway and the findings of this study suggest the feasibility and utility of including human milk in food surveillance systems as an indicator of food security and dietary quality. The annual per capita figure of human milk production per child is of particular value, as this indicator creates a novel basis for evaluating trends within a country and for comparisons between countries. The proportion of potential supply that is ‘lost’ also indicates the potential for improvement against a defined benchmark of clear relevance to public health nutrition.

While the worth of breastfeeding is far greater than can be measured by the volume of milk provided, the availability of such data in food statistics is important for many reasons relevant to nutrition, health and economic policymakers^(41,45)^. Conversely, the lack of reporting of human milk in food statistics has important strategic consequences, particularly where social changes are taking place^(45)^.

The production of human milk by a nation’s mothers can be recognised not only as contributing to national food security and resilience, but also to achieving the Sustainable Developmental Goals. Current influential policy documents are calling for healthier and sustainable food systems. As breastfeeding is such a system, we recommend that food systems conceptual frameworks and surveillance systems should give recognition to human milk by including it as a category of food production. This would help raise awareness of this important national and global food resource among key stakeholders such as officials involved in food monitoring and surveillance systems and management and economic statisticians.

## Supplementary material

For supplementary material accompanying this paper visit https://doi.org/10.1017/S1368980022001495. click here to view supplementary material

## References

[1] Baker P , Santos T , Neves PA et al. (2021) First-food systems transformations and the ultra-processing of infant and young child diets: the determinants, dynamics and consequences of the global rise in commercial milk formula consumption. Matern Child Nutr 17, e13097. 33145965 10.1111/mcn.13097 PMC7988871

[2] Smith JP (2015) Markets, breastfeeding and trade in mothers’ milk. Int Breastfeed J 10, 9. 25829943 10.1186/s13006-015-0034-9 PMC4380115

[3] Roberts TJ , Carnahan E & Gakidou E (2013) Can breastfeeding promote child health equity? A comprehensive analysis of breastfeeding patterns across the developing world and what we can learn from them. BMC Med 11, 254. 24305597 10.1186/1741-7015-11-254 PMC3896843

[4] Victora CG , Bahl R , Barros AJ et al. (2016) Breastfeeding in the 21st century: epidemiology, mechanisms, and lifelong effect. Lancet 387, 475–490. 26869575 10.1016/S0140-6736(15)01024-7

[5] Rollins NC , Bhandari N , Hajeebhoy N et al. (2016) Why invest, and what it will take to improve breastfeeding practices? Lancet 387, 491–504. 26869576 10.1016/S0140-6736(15)01044-2

[6] Walters DD , Phan LTH & Mathisen R (2019) The cost of not breastfeeding: global results from a new tool. Health Policy Plan 34, 407–417. 31236559 10.1093/heapol/czz050 PMC6735804

[7] World Health Organization (WHO) (1998) Complementary Feeding of Young Children in Developing Countries: A Review of Current Scientific Knowledge. Geneva: World Health Organization.

[8] World Health Organization/UNICEF (2003) Global Strategy for Infant and Young Child Feeding. Geneva: World Health Organization (WHO) UNICEF.

[9] Neves PAR , Vaz JS , Maia FS et al. (2021) Rates and time trends in the consumption of breastmilk, formula, and animal milk by children younger than 2 years from 2000 to 2019: analysis of 113 countries. Lancet Child Adolesc Health 5, 619–630. 34245677 10.1016/S2352-4642(21)00163-2 PMC8376656

[10] UNICEF (2017) State of the World’s Children. Children in a Digital World. New York: UNICEF.

[11] Rollins N , Minckas N , Jehan F et al. (2021) A public health approach for deciding policy on infant feeding and mother-infant contact in the context of COVID-19. Lancet Glob Health 9, e552–e557. 33631131 10.1016/S2214-109X(20)30538-6 PMC7906661

[12] Baker P , Smith JP , Salmon L et al. (2016) Global trends and patterns of commercial milk-based formula sales: is an unprecedented infant and young child feeding transition underway? Public Health Nutr 19, 2540–2550. 27211798 10.1017/S1368980016001117 PMC10270963

[13] Dadhich JP , Smith JP , Iellamo A et al. (2021) Climate change and infant nutrition: estimates of greenhouse gas emissions from milk formula sold in selected Asia Pacific countries. J Hum Lact 37, 314–322. 33586512 10.1177/0890334421994769

[14] Sinha B , Chowdhury R , Sankar MJ et al. (2015) Interventions to improve breastfeeding outcomes: a systematic review and meta-analysis. Acta Paediatr 104, 114–134. 10.1111/apa.13127 26183031

[15] National Nutrition Council (1994) Utviklingen i norsk kosthold 1994 (Trends in the Norwegian Diet 1994). Oslo: Statens ernæringsråd.

[16] Norwegian Directorate of Health (2020) Utviklingen i norsk kosthold 2020. Matforsyningsstatistikk (Trends in the Norwegian diet 2020. Food Supply Statistics). Oslo: Helsedirektoratet.

[17] Berg A (1973) The Nutrition Factor; Its Role in National Development. Washington, DC: The Brookings Institution.

[18] Oshaug A & Botten G (1994) Human milk in food supply statistics. Food Policy 19, 479–482.

[19] Smith JP (1999) Human milk supply in Australia. Food Policy 24, 71–91.

[20] Skard A , Busterud M , Eide I et al. (1978) Amming i Norge. En utredning om sosiale og praktiske betingelser for amming (Breast Feeding in Norway. A Report on Social and Practical Conditions for Breastfeeding). Oslo: Ministry of Social Affairs.

[21] Vaz JS , Maia MFS , Neves PAR et al. (2021) Monitoring breastfeeding indicators in high-income countries: levels, trends and challenges. Matern Child Nutr 17, e13137. 33405389 10.1111/mcn.13137 PMC8189208

[22] Statistics Norway (2021) Population. Births. https://www.ssb.no/en/statbank/list/fodte (accessed July 2022).

[23] Lande B (2003) Spedkost 6 måneder. Landsomfattende kostholdsundersøkelse blant spedbarn i Norge. Spedkost 1998–1999 (Spedkost 6 Months. Nationwide Dietary Survey among Infants in Norway, Spedkost 1998–1999). Oslo: Sosial- og helsedirektoratet.

[24] Lande B & Andersen L (2005) Spedkost 12 måneder. Landsomfattende kostholdsundersøkelse blant spedbarn i Norge. Spedkost 1998–1999 (Spedkost 12 Months. Nationwide Dietary Survey among Infants in Norway. Spedkost 1998–1999). Rapport 2005. Oslo: Sosial- og helsedirektoratet.

[25] Lande B & Andersen L (2005) Småbarnskost. Kosthold blant 2-åringer. Landsomfattende kostholdsundersøkelse. Småbarnskost 1999 (Småbarnskost. Nationwide Dietary Survey among 2-Year Old Children in Norway. Småbarnskost 1999). Rapport 2005. Oslo: Sosial- og helsedirektoratet.

[26] Kristiansen A , Andersen L & Lande B (2009) Småbarnskost 2 år. Landsomfattende kostholdsundersøkelse blant 2 år gamle barn. Småbarnskost 2007 (Nationwide Dietary Survey among 2-Year-Old Children in Norway). Rapport 2009. Oslo: Helsedirektoratet.

[27] Øverby NC , Kristiansen AL , Andersen LF et al. (2008) Spedkost 6 måneder. Landsomfattende kostholdsundersøkelse blant 6 måneder gamle barn. Spedkost 2006–2007 (Spedkost 6 Months. Nationwide Dietary Survey among 6 Month Old Infants in Norway. Spedkost 2006–2007). Rapport 2008. Oslo: Helsedirektoratet.

[28] Øverby NC , Kristiansen AL , Andersen LF et al. (2009) Spedkost 12 måneder. Landsomfattende kostholdsundersøkelse blant 12 måneder gamle barn. Spedkost 2006–2007 (Spedkost 12 Months. Nationwide Dietary Survey among 12 Month Old Infants in Norway. Spedkost 2006–2007). Rapport 2009. Oslo: Helsedirektoratet.

[29] Paulsen MM , Myhre JB , Andersen LF et al. (2020) Spedkost 3. Landsomfattende undersøkelse av kostholdet blant spedbarn i Norge, 12 måneder (Spedkost 3. Nationwide Dietary Survey among Infants in Norway, Age 12 Months). Rapport 2020. Oslo: Folkehelseinstituttet og Universitetet i Oslo.

[30] Astrup H , Myhre JB , Andersen LF et al. (2020) Småbarnskost 3. Landsomfattende undersøkelse av kostholdet blant 2-åringer i Norge (Småbarnskost 3. Nationwide Dietary Survey among 2-Year-Olds in Norway). Rapport 2020. Oslo: Folkehelseinstituttet og Universitetet i Oslo.

[31] Myhre JB , Andersen LF & Kristiansen AL (2020) Spedkost 3. Landsomfattende undersøkelse av kostholdet blant spedbarn i Norge, 6 måneder (Spedkost 3. Nationwide Dietary Survey among Infants in Norway, Age 6 Months). Rapport 2020. Oslo: Folkehelseinstituttet og Universitetet i Oslo.

[32] Lande B & Helleve A (2014) Amming og spedbarns kosthold. Landsomfattende undersøkelse 2013 (Breastfeeding and Infant Diet. Nationwide Survey in Norway 2013). Rapport 2014. Oslo: Helsedirektoratet.

[33] Butte N , Lopez-Azarcon M & Graza C (2002) Nutrient Adequacy of Exclusive Breastfeeding for the Term Infant during the First 6 Months of Life. Geneva: World Health Organization.

[34] Norwegian Directorate for Health and Social Affairs (2003) Utviklingen i norsk kosthold 2003. Matforsyningsstatistikk og forbruksundersøkelser (Trends in the Norwegian Diet 2003. Food Supply Statistics and Household Consumption Surveys). Oslo: Sosial-og helsedirektoratet.

[35] Norwegian Directorate of Health (2009) Utviklingen i norsk kosthold 2009. Matforsyningsstatistikk og forbruksundersøkelser (Trends in the Norwegian diet 2009. Food Supply Statistics and Household Consumption Surveys). Oslo: Helsedirektoratet.

[36] Norwegian Directorate of Health (2014) Utviklingen i norsk kosthold 2014 Matforsyningsstatistikk og forbruksundersøkelser (Trends in the Norwegian Diet 2014. Food Supply Statistics and Household Consumption Surveys). Oslo: Helsedirektoratet.

[37] Smith JP (2013) ‘Lost milk?’: counting the economic value of breast milk in gross domestic product. J Hum Lact 29, 537–546. 23855027 10.1177/0890334413494827

[38] Development Initiatives (2020) Global Nutrition Report: Action on Equity to End Malnutrition. Bristol, UK: Development Initiatives.

[39] Raza A , Fox EL , Morris SS et al. (2020) Conceptual framework of food systems for children and adolescents. Global Food Secur 27, 100436.

[40] Fanzo J , Haddad L , Schneider KR et al. (2021) Viewpoint: rigorous monitoring is necessary to guide food system transformation in the countdown to the 2030 global goals. Food Policy 104, 102163.

[41] Smith JP & Ingham LH (2005) Mothers’ milk and measures of economic output. Feminist Econ 11, 41–62.

[42] Stiglitz JE , Sen A & Fitoussi JP (2009) The Measurement of Economic Performance and Social Progress Revisited; Reflections and Overview. Paris: French Observatory of Economic Conditions – Economics Research Center.

[43] Gupta A & Khanna K (1999) Economic value of breastfeeding in India. Natl Med J India 12, 123–127. 10492588

[44] Rohde JE (1982) Mother milk and the Indonesian economy: a major national resource. J Trop Pediatr 28, 166–174. 7131621 10.1093/tropej/28.4.166

[45] Hatloy A & Oshaug A (1997) Human milk: an invisible food resource. J Hum Lact 13, 299–305. 9429365 10.1177/089033449701300415

[46] Smith JP (2017) Markets in Mothers’ Milk: Virtue or Vice, Promise or Problem? Making Milk. London: Bloomsbury Publishing Plc.

[47] Aguayo VM , Ross J , Saunero R et al. (2001) Monetary value of breast milk in Bolivia. Rev Panam Salud Publica 10, 249–256. 11715171 10.1590/s1020-49892001001000005

[48] Aguayo VM & Ross J (2002) The monetary value of human milk in Francophone west Africa: a PROFILES analysis for nutrition policy communication. Food Nutr Bull 23, 153–161. 12094665 10.1177/156482650202300204

[49] Ross J , Chunming C , Zhenying F et al. (2001) Calculating the Effects of Malnutrition on Economic Productivity Health and Survival in China Using PROFILES. Washington, DC: The Academy for Educational Development..

[50] Smith JP (2012) Including Household Production in the System of National Accounts (SNA) – Exploring the Implications of Breastfeeding and Human Milk Provision. Paper Prepared for the 32nd General Conference of The International Association for Research in Income and Wealth, August 5–11, 2012. Boston: International Association for Research in Income and Wealth.

[51] Almroth S , Greiner T & Latham MC (1979) Economic importance of breastfeeding. Food Nutr 5, 4–10. 540668

[52] Latorre G , Martinelli D , Guida P et al. (2021) Impact of COVID-19 pandemic lockdown on exclusive breastfeeding in non-infected mothers. Int Breastfeed J 16, 36. 33865408 10.1186/s13006-021-00382-4 PMC8052849

