# Supplementary material for: The contribution of breastfeeding to a healthy, secure and sustainable food system for infants and young children: monitoring mothers’ milk production in the food surveillance system of Norway
Source: Public Health Nutr. 2022 Jul 4;25(10):2693–701. doi: 10.1017/S1368980022001495 (PMC9991838; doi:10.1017/S1368980022001495)
Supplement: Supplementary file 1 [file S1368980022001495sup.zip › S1368980022001495sup001.docx]

**Supplementary Table 1 Data sources on births and dietary surveys used as basis for the estimation of human milk production in Norway**

|  | **Time period** | | | | |
| --- | --- | --- | --- | --- | --- |
|  | **1993** | **1998-99** | **2006-07** | **2013** | **2018-19** |
| **Breastfeeding (0-24 months of age)**  *Proportion of children breastfed (any breastfeeding) each month* | Data from records in community health services in 5 counties (*SYSBARN*) | National dietary surveys among infants (*Spedkost*) and young children (*Småbarnskost*)/Infant diet and breastfeeding survey | | | |
| 0-12 months of age | Data on any breastfeeding at 3, 6, 9 and 12 months^(17)^ | *Spedkost 1*  1998-99^(21, 22)^  Surveys at 6 months 1998 and 12 months 1999 | *Spedkost 2*  2006-07^(25, 26)^  Surveys at 6 months 2006 and 12 months 2007 | *Infant diet and breastfeeding survey* 2013^(30)^  Survey at 12 months 2013 | *Spedkost 3*  2018-19^(27, 29)^  Surveys at 6 months 2018 and 12 months 2019 |
| 13-24 months of age | No data, suggested estimates | *Småbarnskost 1*  1999^(23)^  Survey at 24 months | *Småbarnskost 2*  2007^(24)^  Survey at 24 months | *Småbarnskost 2* 2007^(24)^  Survey at 24 months | *Småbarnskost 3*  2019^(28)^  Survey at 24 months |
| **Births**  *Number of live births ^(20)^* | Approx. 60 000  (1993) | 58 352  (1998) | 58 545  (2006) | 60 255  (2012) | 55 120  (2018) |
