# Supplementary material for: The contribution of breastfeeding to a healthy, secure and sustainable food system for infants and young children: monitoring mothers’ milk production in the food surveillance system of Norway
Source: Public Health Nutr. 2022 Jul 4;25(10):2693–701. doi: 10.1017/S1368980022001495 (PMC9991838; doi:10.1017/S1368980022001495)
Supplement: Supplementary file 1 [file S1368980022001495sup.zip › S1368980022001495sup002.docx]

**Supplementary Table 2 – Comparison of milk intakes in studies estimating human milk production, litres per infant or child**

| **Author (year of study)**/Months of infant age***** | **Smith (2012)** ^(45)^ | **WHO* (2002)** ^(31)^ | **Aguayo* (2001)** ^(42)^ | **Smith (1999)** ^(16)^ | **WHO* (1998)** ^(11)^ | **Hatloy (1997)** ^(40)^ | **National Nutrition Council** ^(17)^ | **Oshaug (1994)** ^(15)^ | **Gupta (1999)** ^(38)^ | **Almroth (1979)** ^(46)^ | **Rohde (1981/82) ^(39)^** | **Berg (1973)** ^(14)^ |
| --- | --- | --- | --- | --- | --- | --- | --- | --- | --- | --- | --- | --- |
| <6 months | 131 | 136 (120) | 130 (113) | 127 | 133 (113) | 137 | 131 | 127 | 110 | 124 | 108 | 155 |
| 6-12 months | 97 | 155 (94) | 112 | 97 | 123 (113) | 93 | 97 | 97 | 91 | 110 | 72 | 91 |
| <12 months | 228 | 291 (214) | 243 (225) | 224 | 256 (226) | 230 | 228 | 224 | 201 | 234 | 180 | 247 |
| 12-24 months | 79 | **-** | 201 | 106 | 195 | 139 | 79 | 106 | 146 | 146 | 108 | 128 |
| <24 months | 307 | **-** | 443 (436) | 331 | 450 (421) | 369 | 307 | 331 | 347 | 380 | 288 | 375 |
| 24-36 months | **-** | **-** | 93 | **-** | **-** | 93 | **-** | **-** | **-** | **-** | 72 | **-** |
| <36 months | **-** | **-** | 536 (518) | **-** | **-** | 462 | **-** | **-** | **-** | **-** | 360 | **-** |

*bracketed figures are for partially breastfed infants

**Some of these figures are presented in the original study, but for others it is derived from data presented in that study.

*** Estimates of milk volumes are based on somewhat different inclusion of age-groups and may have taken into consideration different rates of exclusive breastfeeding/contribution of complementary foods in the diet.
